# Immunofluorescence and image analysis pipeline for *Drosophila* motor neurons

**DOI:** 10.1093/biomethods/bpz010

**Published:** 2019-08-01

**Authors:** Jeremy R Brown, Chanpasith Phongthachit, Mikolaj J Sulkowski

**Affiliations:** 1 Biology Department, Southern Arkansas University, Magnolia, AR, USA; 2 Department of Molecular, Cellular, and Developmental Biology, University of Colorado, Boulder, CO, USA; 3 Biology Department, Southern Connecticut State University, New Haven, CT, USA

## Abstract

The neuromuscular junction (NMJ) of larval *Drosophila* is widely used as a genetic model for basic neuroscience research. The presynaptic side of the NMJ is formed by axon terminals of motor neurons, the soma of which reside in the ventral ganglion of the central nervous system (CNS). Here we describe a streamlined protocol for dissection and immunostaining of the *Drosophila* CNS and NMJ that allows processing of multiple genotypes within a single staining tube. We also present a computer script called Automated Image Analysis with Background Subtraction which facilitates identification of motor nuclei, quantification of pixel intensity, and background subtraction. Together, these techniques provide a pipeline for neuroscientists to compare levels of different biomolecules in motor nuclei. We conclude that these methods should be adaptable to a variety of different cell and tissue types for the improvement of efficiency, reproducibility, and throughput during data quantification.

## Introduction

The neuromuscular junction (NMJ) of *Drosophila* larvae is a popular model system for neuroscience because of its accessibility, reproducibility, capacity for genetic manipulations, and similarity to glutamatergic synapses within mammalian brains (Reviewed in [1]). NMJ synapses consist of a receptor-rich density on the muscle cell separated from a presynaptic active zone by a narrow synaptic cleft [2]. The active zones are located on the axon terminals of motor neurons. The cell bodies of these motor neurons, which have each been named and identified, reside in the ventral ganglion of the central nervous system (CNS) [3].

We have developed an efficient protocol for immunostaining *Drosophila* larval CNS/NMJ and subsequent image analysis via open-source software with high-throughput capabilities. The protocols and plugin presented herein provide a pipeline capable of both expediting and standardizing the analysis of biomolecule accumulation in *Drosophila* larval motor neurons. Although the current plugin is designed specifically for these motor neurons, the robust staining protocol, versatile analysis software, and background subtraction allow for the application of this methodology to potentially diverse immunofluorescence experiments.

A free, open-source image analysis platform, ImageJ, enables end-users to manually quantify pixel intensities from images [4]. Extensive NMJ morphology and immunofluorescence analysis plugins for ImageJ have been described in detail [5–7]. However, there is currently no equivalent publicly available set of protocols and image analysis software for motor nuclei and soma. Here we describe a robust and versatile protocol for immunostaining of larval CNS and open-access software for the automated analysis of immunofluorescence signal intensity.

## Materials and methods

### *Drosophila* stocks and husbandry

*Drosophila* were kept at room temperature in vials of cornmeal, molasses, and yeast medium [8]. To drive expression of Mad in motor neurons (Figs. 5 and 6), *BG380-Gal4* [9] virgins were crossed to UAS-Mad-GFP [10] males. As a control *BG380-Gal4* virgins were crossed with wild-type (Oregon-R) males. To control for larvae crowding, 8–10 females were crossed with 5–7 males per vial and were passed to fresh vials every 3 days. For measuring the effect of pooling samples on staining variance (Supplementary Fig. S4) wild-type animals (Oregon-R) were used.

### Dissection of *Drosophila* larval CNS

Wandering third-instar stage larvae dissected in ice-cold HL-3 saline as described previously [11, 12]. Filets were fixed for 20 min in 4% paraformaldehyde (PFA) diluted in 1XPBS.Filets from larvae of different genotypes were marked with characteristic incisions for later identification (Fig. 2). Filets were washed several times with wash buffer (0.5% Triton X-100 in 1XPBS), and transferred to a 1.5 ml centrifuge tube for staining.

### Immunostaining

Samples were incubated at 4°C overnight in the following primary antibodies diluted in wash buffer: Rat anti-Elav (DSHB Cat# 7E8A10, RRID: AB_528218) at 1: 500, rabbit anti-pSmad3 (Abcam Cat# ab52903, RRID: AB_882596) at 1: 1000, and chicken anti-GFP (Abcam Cat#ab13960, RRID: AB_300798) at 1: 1000. Samples were then washed and incubated in the following secondary antibodies: Goat anti-rat Alexa Fluor 488 (Thermo Fisher Scientific Cat# A-11006, RRID: AB_2534074) (Supplementary Fig. S2), goat-anti-rat Alexa Fluor 647 (Thermo Fisher Scientific Cat # A-21247, RRID: AB_141778) (Figs 5 and 6), goat anti-chicken Alexa Fluor 488 (Thermo Fisher Scientific Cat# A-11039, RRID: AB_2534096) (Figs 5 and 6), and goat anti-rabbit Alexa Fluor 568 (Thermo Fisher Scientific Cat# A-11011, RRID: AB_143157), each at 1: 500 in wash buffer. Samples were washed thoroughly and rinsed in 1XPBS. Brains were mounted in SlowFade Diamond (ThermoFisher Cat#S36972). 

### Imaging and analysis

Images were acquired with a Zeiss 880 or Nikon Ti-2 confocal microscope using a 40× or 63× objective. Images were analyzed with Imaris^®^ (Bitplane) using the ‘spots’ function to find ROIs and record mean center intensity or with Automated Image Analysis with Background Subtraction (AIABS) as described below. To assess user–user variability, the same image set was analyzed three times by each of the authors of this study. All analyses were performed blind to the identity of the samples.

### Development and design of AIABS

The AIABS script was coded in IJ Macro and Java using the ImageJ script editor of Fiji version 2.0.0 [13]. AIABS utilizes ImageJ API functionality, which are available from the National Institutes of Health [4]. The plugin was built with the script editor and released to the Fiji automatic update service and to the GitHub repository [14].

### AIABS workflow

The majority of parameters employed by AIABS to calibrate functionality are input by the user through a dialog box at the start of the script. AIABS accepts images in .tif RGB stack format. Images are split into color channels of red, green, and blue. The selection channel is dictated by the user. Outside the selection area is cleared and a threshold is applied automatically or manually. A particle analysis is performed based on parameters defined by the user. ROIs returned by the particle analysis are then used to generate a background and measurement mask. The result of the ROI manager ‘Measure’ function are used to generate data which is then exported as a .csv to the working directory.

## Results and discussion

### A novel, all-in-one solution for *Drosophila* immunofluorescence

**Figure 1:**
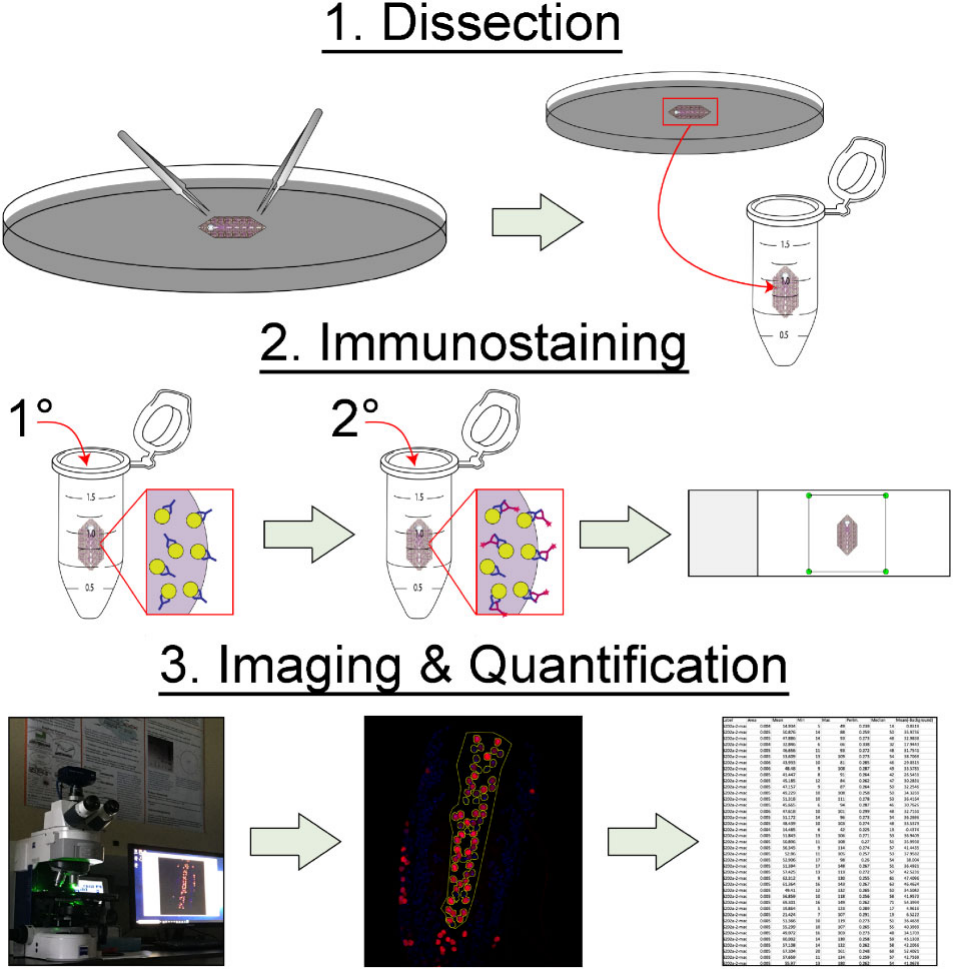
Flow chart and general overview. Graphical overview of the methods presented: 1. The specimen is dissected and then fixed. 2. The specimens are incubated with a primary (1**°**) antibody specific to the target antigen, then a secondary (2**°**) antibody specific to the 1**°** antibody and conjugated to a fluorophore. Excess antibodies are removed by washing and the specimens are mounted on a microscope slide. 3. The specimens are then imaged. Images are analyzed using AIABS, which gathers data from ROIs and subtracts background signals. AIABS compiles data into spreadsheet format for further analysis.

The scientific data pipeline described here consists of a robust and streamlined immunostaining protocol coupled with automated image analysis via an open-access computer program (Fig. 1). For a detailed, step-by-step protocol, see Supplementary Material (Document S1). The immunostaining protocol does not require a blocking step and enables processing multiple control and experimental groups within the same tube. The image analysis program automatically selects regions of interest (ROIs) from one fluorescence channel, superimposes the ROIs onto a different channel, and collects pixel intensity readings from the ROIs. Furthermore, the program measures background immunofluorescence from the region outside the ROIs, subtracts this background from each measurement, and exports data into a .csv spreadsheet document.

### Parallel processing of control and experimental groups

**Figure 2:**
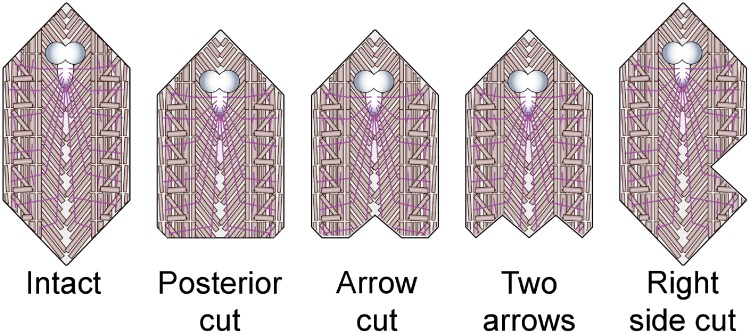
Scoring patterns for identifying specimens. Retaining body wall provides a convenient vehicle for labeling different experimental groups so that staining can be performed in the same tube. It is possible to combine different marking patterns (e.g. arrow and right-side cut) to analyze higher numbers of experimental groups.

An advantage of the presented immunostaining technique is the simultaneous processing of control and experimental groups within the same staining tube. This is made possible by marking individual larval filets with distinctly shaped incisions after fixation (Fig. 2). A potential benefit of this approach is to minimize variables (e.g. washing duration, light exposure, etc.) that could contribute to random errors. In controlled experiments, we saw a slight reduction in variance between samples processed in the same tube compared to samples processed in individual tubes [co-efficient of variance (CV) of 0.261 versus 0.301, respectively], but this difference was not statistically significant (*P* = 0.561) (Supplementary Fig. S2). Even though a difference in variance was not apparent in controlled experiments, it is good practice to minimize variables, and processing samples simultaneously could prevent occasional blunders from generating spurious results.

To mark the samples, the larval filet technique must be used, which requires some practice to achieve proficiency but provides the additional benefit of staining NMJs, which can then be imaged from the same specimen. If the aim is simply to obtain CNS, filets do not have to be perfect. Alternative dissection techniques, such as inverting the larva [15], are easier, but unfortunately render it impossible to mark samples in the way described above. Another downside of the “inside-out” technique is that mounting CNS becomes more difficult and laborious as the CNS must be dissected away from the carcass after staining.

### Blocking is unnecessary for *Drosophila* immunofluorescence

Immunostaining protocols routinely call for incubating samples in serum, albumin, or other reagents to prevent non-specific binding of primary and secondary antibodies. Another advantage of our staining method is that it does not require a blocking step, or that antibodies be diluted in blocking buffer. We have not noticed any benefit from blocking on staining CNS or NMJ tissues with a variety of antibodies. All steps of the immunostaining procedure are performed using the same washing buffer (1XPBS + 0.5% Triton X-100), which further streamlines and simplifies the procedure. Our results are consistent with a recent study that found no effect from blocking on immunostaining of mammalian cells or tissue samples [16].

### Standardization limitations and leeway

**Figure 3:**
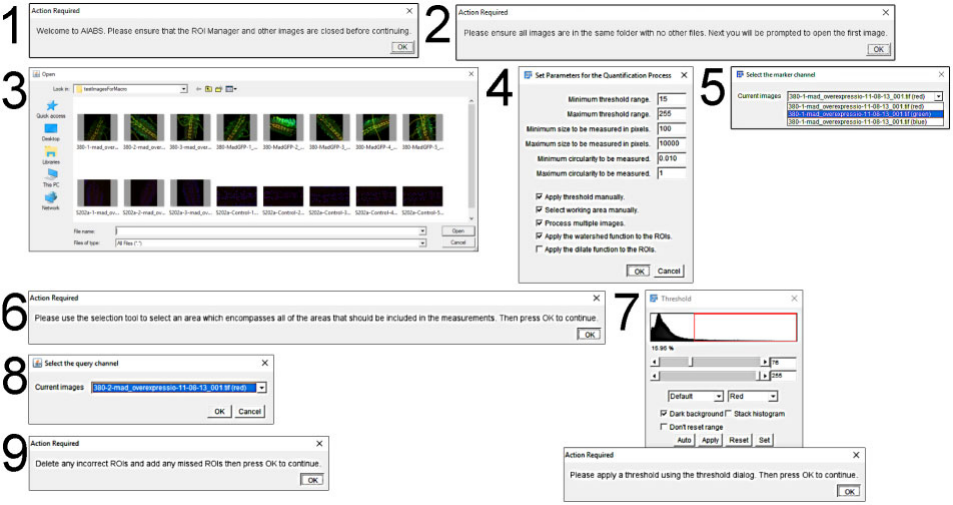
Plugin graphical user interface. Steps 1 and 2 ensure that the system is setup properly. Step 3 prompts the user to select the first image to be analyzed and then press ‘Open’. Step 4 is the main dialog box for inputting parameters. Step 5 instructs the user to select the marker channel. Step 6 directs the user to draw a selection area. Step 7 directs the user to apply a threshold. Step 8 allows the user to select the query channel. Step 9 is a dialogue box that waits for the user to inspect and correct ROIs. For more information regarding plugin operation, please see Supplementary Movie S3, which demonstrates this process.

For standardization, care must be taken when imaging to maintain all parameters (laser intensity, gain, etc.) identically between experimental and control groups. Furthermore, all pixel intensities should be maintained within the dynamic range, avoiding zero signals or saturation. Our methodology helps in maintaining signals within the dynamic range because the use of an independent masking, or marker, channel (e.g. Elav) makes it unnecessary to have the experimental channel exposed to a level that would allow it to function as a marker (Fig. 3). One potential limitation of our methodology is the reliance on Elav staining for identifying motor neurons. This can be problematic if the experiment requires another rat antibody. One could employ the use of genetically encoded markers, such as an enhancer-trap for the glutamate vesicular transporter *VGLUT-GAL4,* to solve this problem [17]. However, this would require all control and experimental groups to carry these transgenes.

### Manual optimization requires minimal effort with AIABS

**Figure 4:**
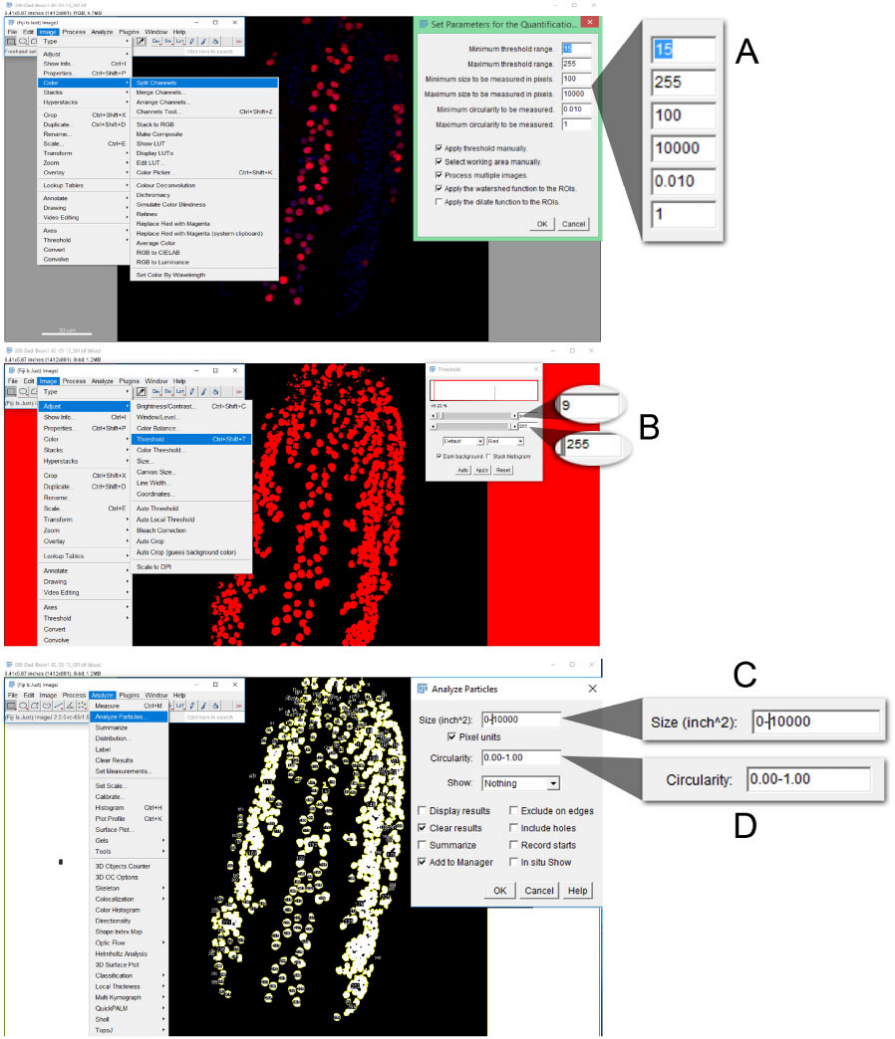
Optimizing plugin parameters. (**A**) The plugin initially offers default values for parameters that work well for motor neuron immunofluorescence. (**B**) If the “Apply threshold manually” box is checked, AIABS allows the user to adjust thresholds using the min/max sliders. (**C–D**) To manually adjust particle properties try different settings with the “Analyze Particles” function (Analyze->Analyze Particles). To test the effect of watershed or dilate, apply the desired function after the threshold has been applied but before the particle analysis.

Once the plugin is installed and the user is comfortable with the interface, AIABS can be standardized and automated following optimization of the plugin parameters (Fig. 4). For our images of larval motor neuron immunofluorescence, the default parameters generally do not require modification. However, depending on the size of the objects to be analyzed, the resolution of the image, and intensity, optimum parameters may have to be empirically determined. Notice that the threshold can be set in the parameters dialog (Fig. 4A), which provides a starting value. AIABS includes parameter persistence, which means that values entered into the parameters dialogue box will be retained for subsequent runs. The threshold can then be optimized further by using the sliders (Fig. 4B), which allow the user to continuously visualize the effects of the threshold levels.

### AIABS generates results comparable to commercial solutions

**Figure 5:**
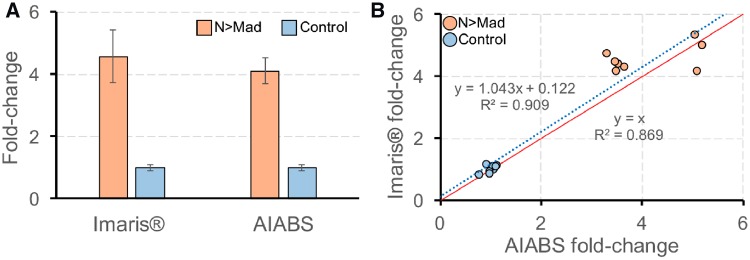
AIABS generates similar intensity measurements compared with commercial image-analysis software. (**A**) Mean fold-change of pMad immunofluorescence in motor nuclei of animals overexpressing Mad-GFP in motor neurons (N>Mad) compared to controls as measured by Imaris^®^ software and AIABS. The same image set of eight CNS of each genotype (*n* = 8) was used for each analysis. Error bars indicate SD. (**B**) Fold difference of pMad immunofluorescence in individual images from the same set measured with Imaris^®^ and AIABS. Linear regression best fit is shown as dotted blue line. Solid red line shows a 1: 1 relationship (*y* = *x*). The difference in fold-change was not significant per Student’s *t*-test (*P* = 0.315).

In order to test that the data generated by AIABS accurately reflect immunostaining intensity, we compared data obtained by analyzing the same image set with AIABS and with Imaris^®^ software (Bitplane) (Fig. 5). The image set consisted of eight CNS from animals overexpressing Mad-GFP in motor neurons and eight controls from a previous study [18]. The “spots” feature of Imaris^®^ was used to identify pMad-positive motor nuclei and mean center intensity was recorded, as described previously [18, 19]. The mean fold-change in pMad immunofluorescence intensity resulting from Mad overexpression measured by Imaris was 4.56 ± 0.4, whereas AIABS measured 4.1 ± 0.09 (Fig. 5A). There was a strong correlation between individual readings obtained with Imaris^®^ and AIABS (*R*^2^^ ^= 0.909) (Fig. 5B). The reason for the difference between the two programs could result from the absence of a masking channel with Imaris. Therefore, AIABS could have detected some motor neurons with lower levels of pMad. Overall, the agreement between the datasets shows that AIABS is suitable for quantification of immunofluorescence intensity.

### Minimal user–user variability with AIABS

**Figure 6:**
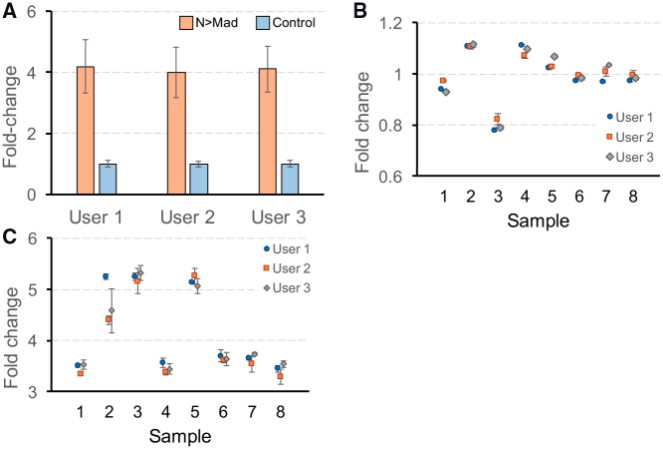
AIABS shows minimal user-to-user variability. (**A**) Fold-change of pMad immunofluorescence in motor nuclei of CNS from eight animals overexpressing Mad-GFP in motor neurons (N>Mad) (*n* = 8) compared to eight control animals (*n* = 8) as measured by three different users. Each user measured each of the samples three times. (**B**) Fold-change of each of the control samples measured three times by each user. (**C**) Fold-change of each of the N>Mad samples measured three times by each user. Error bars in each panel display SD. Differences in fold-changes between users not significant per ANOVA (*P* = 0.902).

A major goal of quantitative image analysis is removing subjectivity and increasing reproducibility. We predict that the automated ROI identification and background subtraction of AIABS will minimize user–user variability. To test this prediction, three users analyzed the same set of sixteen images that was used for Fig. 5 three times each (Fig. 6). The fold-change of pMad immunofluorescence following Mad-GFP overexpression measured by the three users was 4.187 ± 0.877, 4.001 ± 0.824, and 4.106 ± 0.761. The differences between the users were not statistically significant (*P* = 0.902). Thus, AIABS shows minimal user–user variability, which can assist in comparing data within labs and among research groups.

AIABS relies on a modeling technique which uses binary decisions such as thresholding for feature extraction. The threshold is not entirely objective as the user ultimately makes that decision based on knowledge and experience. Data quality depends on many factors including the skill of the experimenter in dissection, mounting, immunohistochemistry, and fluorescent imaging, as well as the quality of the instrumentation used. A comparison of different thresholding models shows many effects resulting from threshold choice [20]. We found that despite these effects three individuals replicated similar results when quantifying the provided dataset using AIABS (Fig. 6). Although the threshold step is subjective it does appear to produce reproducible results when applied by individuals that have experience with the data type.

### Comparison of AIABS to other ImageJ plugins

There are plugins for ImageJ which perform automated signal quantification but to our knowledge only manual quantification of immunofluorescence has been documented [21]. EzColocalization (EzCo) is another open-source tool recently developed for ImageJ that can be used to quantify immunofluorescence data [22]. EzCo quantifies colocalization and so has multiple marker channels instead of a single marker like AIABS. Compared to AIABS, EzCo has more customizability and includes advanced features such as custom scripting. EzCo also has more robust built-in analytics including heat maps and scatterplots. AIABS on the other hand has a more streamlined and accessible interface that is specifically designed to obtain intensity measurements. There are other data extraction solutions for fluorescent microscopy such as PixFRET, ThunderSTORM [23, 24]. In contrast to these options, AIABS is tailored specifically for ease of automated immunofluorescence data extraction. We believe that the ease of use of AIABS will place image quantification within the reach of researchers with minimal experience, and can even be used in educational settings.

### A solution for data extraction from *Drosophila*

The methods presented here provide a comprehensive set of instructions to guide researchers through all the steps of obtaining quantitative data from *Drosophila* motor neurons. The optimized immunostaining protocol and novel sample identification technique can benefit novice and experienced researchers alike. The AIABS ImageJ plugin is a versatile open-source software that identifies ROIs from a subset of the image, subtracts the area outside of the ROIs as background, and exports results in spreadsheet format. AIABS can process batches of images, making it ideal for high-throughput applications like genetic screens.

Although these methods have been optimized for *Drosophila* CNS and motor neurons, we see no reason why they could not be applicable to a variety of different tissue and cell types. This is due to the fact that this methodology uses general computer vision and algorithmic feature extraction with techniques such as binary thresholding and particle analysis. In the future, more features such as z-stack to voxel conversion may be implemented by the current developer, or by others who decide to modify and contribute to this script.

## Supplementary Material

bpz010_Supplementary_Data

## Data Availability

All data used for analyses are available in the supplementary material.
