# Supplementary material for: Immunofluorescence and image analysis pipeline for Drosophila motor neurons
Source: Biol Methods Protoc. 2019 Aug 1;4(1):bpz010. doi: 10.1093/biomethods/bpz010 (PMC6676502; doi:10.1093/biomethods/bpz010)
Supplement: bpz010_Supplementary_Data [file bpz010_supplementary_data.zip › DocumentS1.docx]

**Supplementary Information:**

**Detailed protocol:**

1. **Dissection of *Drosophila* larval CNS**
   1. Isolate individual wandering third instar larva from vial. Wash surface debri from larvae by immersing in water or 1XPBS.
   2. Transfer larva to 1.5 ml centrifuge tube containing ice-cold HL-3 saline [[8]](https://www.zotero.org/google-docs/?jyBpQ2). Place tube in ice bath for 30 seconds to anaesthetize larvae.
   3. Use #5 forceps to transfer larva from centrifuge tube to Silgard plate. Use forceps to move ice-cold HL-3 onto larva to form droplet of ~200 µl.
   4. Insert minutiens pin (0.10 mm diameter) through posterior of larva (in between posterior spiracles) to fasten larva to Silgard plate.
   5. Insert minutiens pin through anterior of larva (just posterior to mouth hooks). Use pin to stretch larva to full extension (don’t overstretch) and attach to Silgard plate.
   6. Replace HL-3 from larva with fresh ice-cold HL-3.
   7. Use scissors to make incision along frontal plane at posterior of larva to allow scissors to enter.
   8. Cut from incision to anterior pin along dorsal midline.
   9. Remove HL-3 from larva. Perfuse body cavity several times with fresh, ice-cold HL-3. Replace this HL-3 with fresh, ice-cold HL-3.
   10. Use forceps to remove larval viscera. Be careful not to damage CNS. Siphon out viscera and replace with fresh ice-cold HL-3.
   11. Insert four additional minutiens pins to attach body wall to Silgard plate.
   12. Replace HL-3 with 200 µl of 4% para-formaldehyde (PFA) in 1XPBS or other fixative (e.g. Bouin’s fixative).
   13. Fix for 20 minutes.

**NOTE:** Actual fixation time depends on fixative,  antibody, and tissue type, and should be optimized (e.g. fixation in Bouin’s takes 2 minutes for larval filets)

- 1. Replace PFA with 300 µl 1XPBS. Replace 1XPBS to rinse.

1. **Immunohistochemistry**
   1. Remove pins from fixed larval filet.
   2. Use forceps to transfer filets to 1.5 ml centrifuge tube containing 1 ml of wash buffer (0.5% Triton X-100 in 1XPBS).
   3. Siphon off the wash buffer taking care to not damage the samples.
   4. Fill tube with fresh wash buffer.
   5. Repeat wash steps 2.3 and 2.4 at least three times before moving on.
   6. Replace the wash buffer with 500 μl of primary antibodies diluted in wash buffer. Rat anti-Elav (DSHB Cat# Rat-Elav-7E8A10 anti-elav, RRID:AB_528218) is used at 1:500 (1 μl). Rabbit anti-pSmad (Abcam Cat# ab52903, RRID:AB_882596) were used at 1:1000 (0.5 μl).

**NOTE:** We have found blocking with serum unnecessary for *Drosophila* tissues. For other tissue types and antibodies, please test whether blocking helps reduce background.

Incubate samples in primary antibody at 4**°**C overnight.

1. Wash samples by replacing primary antibody with wash buffer. Invert to mix and incubate at room temperature for ~15 minutes. Repeat this wash step two more times for a total of three washes.

**NOTE:** Primary antibody can be stored with 0.05% sodium azide and reused several times.

1. Replace the wash buffer with 500 μl of secondary antibody diluted in wash buffer. Goat anti-Rat Alexa Fluor 488 (Thermo Fisher Scientific Cat# A-11006, RRID:AB_2534074) and Goat anti-Rabbit Alexa Fluor 568 (Thermo Fisher Scientific Cat# A-11011, RRID:AB_143157) were used at 1:500 (1 μl).

**NOTE:** From this step forward, avoid exposing samples to light to prevent photobleaching of fluorophores.

1. Incubate samples in secondary antibody at 4**°**C overnight.
2. Wash samples by replacing secondary antibody with wash buffer. Invert to mix and incubate at room temperature for ~15 minutes. Repeat this wash step four more times for a total of five washes.
3. Replace wash buffer with 500 μl of 1XPBS.
4. Decant the samples into a depression of a 9-well spot plate.
5. Fill one well of plate with 100 μl of mountant (e.g. SlowFade Diamond, ThermoFisher Cat#S36972) into an empty depressions in the 9-well spot plate.
6. Use forceps to carefully remove the CNS from the carcass and transfer the CNS into the well containing the mountant. Repeat until all CNS are in the mountant well. Keep CNS in mountant 5 minutes to equilibrate.
7. Use forceps to transfer the brains onto a clean microscope slide.
8. Arrange the CNS and orient such that ventral side is facing up using 22-gauge syringe needles.
9. Use a clean coverslip to scrape a small dot of modeling clay onto each corner of the coverslip.
10. Place the coverslip over the brains taking care not to move them out of orientation.
11. Press down until samples contact coverslip, taking care not to crush samples.
12. Use micropipette to inject mountant to replace the gap of air between the coverslip and the slide via capillary action.
13. Seal the edges of the coverslip with nail polish.
14. Use marker to draw circles around the samples on the bottom of the slide.
15. Image the sample.
16. **Fluorescence intensity quantification**
17. Download and install AIABS.
    1. Download and install Fiji/ImageJ from: <https://imagej.net/Fiji/Downloads>

1.2 To install AIABS with automatic updates follow the instructions in this link: <https://imagej.net/Following_an_update_site>

AIABS update site url:

<http://sites.imagej.net/AIABS>

**NOTE:** To manually download AIABS follow the instructions in this link: <https://github.com/jrb07/AIABS>

1. Process images.
   1. Open the ‘Plugins’ dropdown.
   2. Select ‘AIABS’, then press ‘OK’.
   3. When prompted, open the first image to be quantified.

**NOTE:** AIABS will not process batches correctly if there are files other than .tif images in the directory.

- 1. Assign the parameters for AIABS using the dialog box. (See Fig.4 for parameter optimization.)
  2. Select the color channel of your marker when prompted using the drop-down menu.

**NOTE:** The script will save your choice and automatically select the same color channel in subsequent runs.

- 1. If manual selection is enabled then draw around the neuropil using the selection tool before pressing OK. Otherwise, the script will not proceed.
  2. If manual thresholding is enabled, set the threshold values then press apply before pressing OK. If not then skip this step.
  3. Review the ROIs, delete any incorrect ROIs, and add any missing ROIs with various selection tools.Press OK when done.

**NOTE:** Splitting ROIs into individual sub-ROIs can be done at this step by holding alt while using any selection tool to make a composite selection and then use the ROI Manager’s ‘Split’ function.

- 1. Select the color channel that is to be measured when prompted using the drop-down menu.

**NOTE:** The script will select the same color channel automatically in subsequent runs.

- 1. Repeat steps 2.6 - 2.9 until there are no remaining images.
