# Supplementary material for: Immunofluorescence and image analysis pipeline for Drosophila motor neurons
Source: Biol Methods Protoc. 2019 Aug 1;4(1):bpz010. doi: 10.1093/biomethods/bpz010 (PMC6676502; doi:10.1093/biomethods/bpz010)
Supplement: bpz010_Supplementary_Data [file bpz010_supplementary_data.zip › Figure S2.docx]

Figure S2. Effect on variance of staining samples in separate tubes compared with staining samples in the same tube. Bars represent mean CV of pMad immunofluorescence of three experiments in which 6-10 larvae were stained either in the same tube or with each sample in a separate tube. Difference is not significant per Student’s t-test (p=0.561).
